# Evidence for Bone Marrow Adult Stem Cell Plasticity: Properties, Molecular Mechanisms, Negative Aspects, and Clinical Applications of Hematopoietic and Mesenchymal Stem Cells Transdifferentiation

**DOI:** 10.1155/2013/589139

**Published:** 2013-03-27

**Authors:** Ivana Catacchio, Simona Berardi, Antonia Reale, Annunziata De Luisi, Vito Racanelli, Angelo Vacca, Roberto Ria

**Affiliations:** ^1^Department of Biomedical Sciences and Human Oncology, Section of Internal Medicine and Clinical Oncology, University of Bari Medical School, Policlinico, Piazza Giulio Cesare 11, I-70124 Bari, Italy; ^2^Department of Biomedical Sciences and Human Oncology, Section of Internal Medicine, University of Bari Medical School, Policlinico, Piazza Giulio Cesare 11, I-70124 Bari, Italy

## Abstract

In contrast to the pluripotent *embryonic stem cells* (ESCs) which are able to give rise to all cell types of the body, mammalian *adult stem cells* (ASCs) appeared to be more limited in their differentiation potential and to be committed to their tissue of origin. Recently, surprising new findings have contradicted central dogmas of commitment of ASCs by showing their plasticity to differentiate across tissue lineage boundaries, irrespective of classical germ layer designations. The present paper supports the plasticity of the *bone marrow stem cells* (BMSCs), bringing the most striking and the latest evidences of the transdifferentiation properties of the *bone marrow hematopoietic and mesenchymal stem cells* (BMHSCs, and BMMSCs), the two BM populations of ASCs better characterized. In addition, we report the possible mechanisms that may explain these events, outlining the clinical importance of these phenomena and the relative problems.

## 1. Introduction

### 1.1. Evidence for BMSCs Plasticity

 It has long been believed that the differentiation potential of ASCs is restricted to the production of the cell types normally found in the organ in which ASCs reside. Classical experiments showed that when fragments or cells dissociated from an organ or a tissue are transplanted to a new site or cultured, they tend to maintain their originalcharacter; although they may lose some of their properties, they usually do not acquire characteristics of a different cell lineage [1]. The first suggestion that ASCs, committed to a specific developmental lineage, switch into another cell type of an unrelated tissue (transdifferentiation) came from studies of whole BM transplantation in humans and animal models. In 1997 Eglitis and Mezey reported that transplanted mouse BM cells could give rise to brain astrocytes in adult mice [2]. The most striking suggestion of stem cell plasticity was published in 1998 by an Italian group, which found that mouse BM cells could give rise to skeletal muscle cells when transplanted into a mouse muscle that had been damaged by an injection of a muscle toxin [3]; thus mouse BMSCs could migrate to sites of muscle injury and participate in muscle regeneration, albeit at low efficiency. From 1999 up to date it was reported that transplanted BM cells could produce hepatocytes [4–7], endothelial [8] and myocardial cells [9–11], central nervous system (CNS) neurons, and glial cells [12–14]. The reason why these forms of plasticity were not been seen before is probably due to the methods used. In earlier experiments, organ or tissue fragments were usually transplanted, and so the donor cells continued to have neighbors of the same tissue type. In the subsequent experiments, cell suspensions were usually transplanted so that individual donor cells could end up surrounded by cells of a different tissue type. Moreover, the donor cells were genetically marked so that even rare cells expressing donor cell genes could be identified in tissue sections. Sex chromosome markers (Y chromosome DNA sequences to detect male donor-derived cells in female hosts) have been used to detect plasticity in BM transplant patients, where BM or blood cells were reported to give rise to either hepatocytes [15, 16] or epithelial cells in skin and gut [16]. 

These and similar studies, performed with transplanted BM cells, suggested that BM is a source of different kinds of ASCs which, given the appropriate environmental signals, show pluripotent properties and transdifferentiate into cells of many different organs, including skeletal muscle, heart, liver, and endothelial and even brain cells. 

Our focus is to critically evaluate the evidence in favor of HSCs and MSCs plasticity. 

### 1.2. From Multipotent to Pluripotent BMHSC

**Figure 1:**
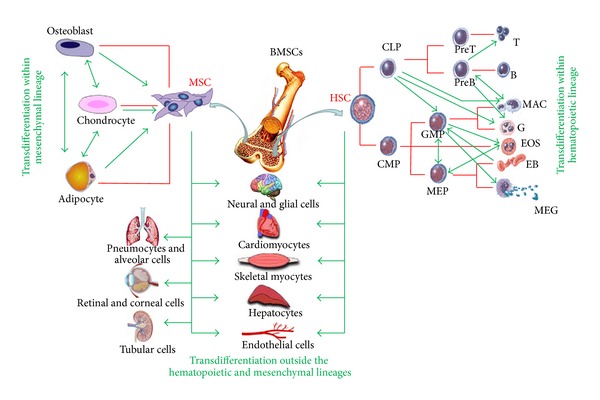
Plasticity of BM HSC and MSC.

HSCs are essential for the generation and homeostasis of the blood system. They give rise to all the blood cell types, including lymphocytes, erythrocytes, monocytes, granulocytes, and platelets, and they replenish these cells [17] (Figure 1). Contrary to ASCs from other tissues, HSCs are easy to obtain, as they can be either aspirated directly out of the BM or stimulated to move into the peripheral blood (PB) stream, where they can easily be collected. According to the hierarchy of hematopoietic development, an HSC would be positioned at a branch bifurcation with its potential restricted to generating *common lymphoid precursors* (CLPs) [18] and *common myeloid precursors* (CMPs) [19]. 

#### 1.2.1. Transdifferentiation of BMHSCs into Nonhematopoietic Cells

To support the hypothesis that HSCs are able to transdifferentiate into nonhematopoietic cells (Figure 1), several groups transplanted purified BMHSCs in a variety of settings. Gussoni et al. transplanted HSCs from male mice into female mdx mice, a model of Duchenne muscular dystrophy [20]. They were able to track the fate of the transplanted cells by detecting the Y chromosome with fluorescent in situ hybridization. The donor cells efficiently replenished the BM of the recipients as expected, and cells from the males expressing dystrophin were found at low levels in host muscle fibres, indicating differentiation of the transplanted cells into muscle. Analogous studies have shown that HSCs can also contribute to the repair of capillaries and cardiomyocytes in a mouse model of coronary artery infarction [21]. Orlic et al. observed that when a population enriched in HSCs was injected directly into injured hearts, it could participate in the regeneration of cardiac muscle, leading to an apparent improvement of cardiac function [9]. Lagasse et al. also supported the concept of transdifferentiation at functional level. They showed that HSCs injected into mice with an inducible lethal liver disease, tyrosinemia type 1, could repopulate the haematopoietic system as well as differentiate into hepatocytes and rescue the animals from hepatic failure and death [22]. 

#### 1.2.2. Transdifferentiation of BM HSCs within the Hematopoietic System

Over the past two decades, results from *in vitro *studies have challenged the notion of a strictly hierarchical branching model of hematopoiesis. Numerous investigations have shown that both nontransformed and malignant hematopoietic precursors can switch cell types within the hematopoietic lineage [23] (Figure 1). Many distinct lymphoid-to-myeloid and myeloid-to-erythroid switches were shown by inducing transcription factor expression, cytokine or drug treatments, and changes in environmental conditions [24, 25] (Figure 1). The first experiment demonstrating switch between lymphoid and myeloid cells was conducted by Boyd and Schrader, who tested the effects of 5-azacytidine on Abelson virus-transformed pre-B lymphoma cell lines. They found that a subset of these cells acquired properties of macrophages [26]. A similar effect was seen in pre-B and B-cell lines immortalized with *Eu-myc*, in which Klinken et al. overexpressed the *v-raf *oncogene. The *v-raf*-transfected cells not only expressed myelomonocytic markers (such as the *colony-stimulating factor* (CSF)-1 receptor and lysozyme), but also retained immunoglobulin rearrangements characteristic of the original cells [27]. Similarly, a proportion of early B-cell lines ectopically expressing the *v-fms *oncogene (encoding a constitutively active form of the CSF-1 receptor) switched into macrophages [28]. Moreover a study reported a switch of B-lymphoid cells to neutrophil granulocytes [29]. A surprising degree of plasticity was discovered in B-lineage cells derived from Pax5 knockout mice: in the absence of Pax5, commitment of lymphoid progenitor to the B-lymphoid lineage was blocked and pre-B cells from mice, carrying a deletion in the Pax5 gene, could generate multilineage hematopoietic cells [30]. It appears that the main role of Pax5 in the establishment of B-cell commitment is the repression of lineage inappropriate genes, such as the CSF-1 receptor gene (*c-fms*), which is expressed in the pre-B Pax5 knockout cells. Another series of experiments demonstrated that CLPs can be reprogrammed to become myelomonocytic and that lineage plasticity has been observed also within the myeloid/erythroid compartment [23].

### 1.3. From Multipotent to Pluripotent BMMSC

MSCs isolated from the BM of adult organisms were initially characterized as plastic adherent, fibroblastoid cells with the capacity to differentiate into osteoblasts, adipocytes, and chondrocytes *in vitro* (Figure 1) and in to heterotopic osseous tissue when transplanted *in vivo* [31]. In addition to BM, MSCs have also been elaborated from skeletal muscle, adipose tissue, umbilical cord, synovium, the circulatory system, dental pulp, and amniotic fluid as well as fetal blood, BM, liver, and lung [32]. Therefore, it appears that MSCs reside within the connective tissue of most organs as predicted by early studies with chick embryos [32]. However, it should be noted that these populations are not functionally equivalent with respect to their *in vivo* differentiation potential [33]. Despite their functional heterogeneity, MSCs populations obtained from most tissues commonly express a number of surface receptors including CD29, CD44, CD49a-f, CD51, CD73, CD105, CD106, CD166, and Stro-1 and lack expression of definitive hematopoietic lineage markers including CD11b, CD14, and CD45 [33]. However, it is important to realize that no single isolation method is regarded as a standard in the field. Therefore, the varied approaches used to culture, expand, and select MSCs make it difficult to directly compare experimental results.

#### 1.3.1. Transdifferentiation of BMMSCs in Non-Mesenchymal Cells

Kopen et al. first demonstrated that MSCs injected into the CNS of newborn mice migrated throughout the brain and adopted morphological and phenotypic characteristics of astrocytes and neurons [33]. These findings were confirmed by other laboratories [34, 35], which tried to identify the conditions that induced neural differentiation of MSCs *in vitro*. Several groups reported that exposure to reducing agents and antioxidants or chemicals, that increase intracellular cyclic AMP levels, induced MSCs to adopt a neuron-like morphology and express various neural specific proteins including nestin, *glial fibrillary acidic protein* (GFAP), *neurofilament heavy chain *(NF-HC), and *β*-III tubulin [36, 37]. Studies by Neuhuber et al. [38] showed that these agents promoted retraction of the cell cytoplasm due to disruption of the actin network in MSCs and not neurite outgrowth as seen in neurons. Microarray [39] and proteomic studies [40] further demonstrated that the set of genes modulated in MSCs after neural differentiation was distinct from the set differentially expressed between untreated MSCs and neural tissue. Therefore, cytoskeletal alterations induced by these agents rather then transdifferentiation accounted for the neuron-like morphology of MSCs. Moreover BM is also innervated by nervous tissue, which explains the finding that MSCs from BM also express various neuroregulatory proteins including neurotrophins, neurite-inducing factors, and neuropeptides. Surprisingly in 2008 Tondreau et al. reported that BM MSCs have the potential to differentiate in to neuronal cells with specific gene expression and functional properties [41]. More recently it has been reported that BMMSCs possess a great potential to differentiate into functional neurons because they not only expressed neuron phenotype and membrane channel protein, but also exhibited functional ion currents [42]. Thus evidence for transdifferentiation of BMMSCs into neurons is contradictory.

BMMSCs have also been reported to differentiate into various epithelial cell types after systemic administration *in vivo*. It was shown that BMMSCs engraftment in lung of mice was enhanced in response to bleomycin exposure and that a small percentage of MSCs, localized to areas of lung injury, resembled epithelial cells and copurified with type II pneumocytes [32]. Moreover BMMSCs engrafted in lung differentiated into type I pneumocytes or assumed phenotypic characteristics of all major cell types in lung including fibroblasts, type I and type II epithelial cells, and myofibroblasts [32]. Recently it has been reported that BMMSCs can differentiate into type II alveolar epithelial cells *in vitro* [43]. BMMSCs can also differentiate into skin epithelial cells, sebaceous duct cells [44], retinal pigment epithelial cells [45], corneal keratocytes phenotype [46], and tubular epithelial cells [47]. 

MSCs can also differentiate and integrate into muscle cells [48]. A recent report supports the myogenic potential of BMMSCs *in vitro* and *in vivo* for the treatment of urinary incontinence [49]. MSCs appear also to be involved in the generation of myocardial cell types [50]. Not only expression of some genes indicative of myocardial differentiation like troponin T, **β*-myosin heavy chain* (*β*MHC), *myosin regulatory ligh chain 2* (Myl 2) but also detailed analysis of contractility, excitation-contraction coupling and signalling pathways demonstrated that MSCs can generate functional cardiomyocytes *in vitro* [51].

Transdifferentiation of cultured naïve MSCs into hepatocyte-like cells has been claimed to occur by adding specific differentiation media [52]. Recently Zhang et al. have resumed the factors and the methods used to differentiate MSCs, from BM and other tissues, into hepatocyte-like cells underlying their liver regenerative potential [53]. However in many transplantation experiments naïve or differentiated murine or human MSCs were not able to generate liver tissue and to rescue the liver phenotype in an *albumin-urokinase promoter* (Alb-uPA) transgenic mice or in *fumarylacetoacetate-hydrolase*-(FAH-)-deficient mice (FAH^(−/−)^). Transplantation of BMMSCs-derived hepatocyte-like cells into a patient with homozygous familial hypercholesterolemia failed to affect the cholesterol levels [54]. Evidence from the literature also points towards protective and trophic effects of MSCs when injected into the injured liver but the exact therapeutic mechanisms are unknown [55]. 

#### 1.3.2. Transdifferentiation of BMMSCs within Mesenchymal System

Similar to transdifferentiation observed in the hematopoietic system (lymphoid-to-myeloid and myeloid-to-erythroid switches), transdifferentiation examples have also been reported in mesenchymal system (Figure 1). Song and Tuan reported that fully differentiated osteoblasts, from BMMSCs, were able to change their differentiation program and became lipid-producing adipocytes and chondrocytes that produced proteoglycan, collagen type II, and link protein [56]. They also demonstrated that human MSCs that had differentiated into adipocytes transdifferentiated into osteoblasts or chondrocytes by replacing the inducing culture media. Similarly, chondrocytes derived from MSCs in the presence of TGF-*β*3 could be induced to differentiate into osteoblasts and adipocytes [56]. In the same report the authors showed that without the pressure of inducing factors, fully differentiated MSC-derived cells could resume cell proliferation, modify their gene expression profile, and return to a more primitive stem cell-like stage. Accompanying the phenotypic changes observed, was a fluctuation in the expression of lineage-specific transcription factors: Cbfa 1 for osteogenesis, Sox 9 for chondrogenesis, and PPAR*γ*2 for adipogenesis. As expected, expression of Cbfa 1 was upregulated during osteogenesis, whereas both Sox 9 and PPAR*γ*2 were downregulated compared with undifferentiated human MSCs. On the other hand, expression levels of all three transcription factors decreased during osteoblast dedifferentiation, which suggested that cells might return to an uncommitted developmental stage from a fully determined cell type (dedifferentiation) [56]. Thus differentiation processes are not unidirectional as regarded for a long time; dedifferentiation of committed progenitors and successive differentiation in other cell types are possible, at least for mesenchymal and hematopoietic system. It remains to be determined whether dedifferentiation of committed progenitors is only an experimentally induced effect or whether this is also taking place normally under physiological conditions. 

The red lines indicate normal lineage relationships, and the thick green lines represent transdifferentiation within and outside the hematopoietic and mesenchymal lineages. (These switches do not necessary imply direct transitions.) 

### 1.4. Mechanisms Underlying BM HSC and MSC Plasticity

#### 1.4.1. Microenvironment-Dependent Reprogramming of Gene Expression Profile Underlying Transdifferentiation in HSCs and MSCs

In order to undergo transdifferentiation and fate changes compared to their own lineage commitment, ASCs need to change or modify their gene expression programs. Therefore, temporary inactivation of cellular memory of transcriptional state is required. It is well known that BM microenvironment, in which HSCs and MSCs reside, provides signals for survival and external control of stem cell activity. In this regard it can be assumed that new microenvironment signals should be able to modulate the cellular memory of transcriptional state and to lead to a switch in stem cell gene expression and in its cellular identity. Transplanted ASCs may recognise heterotopic environments through cell surface receptors, which stimulate signaling transduction pathways connecting the outside of the stem cell with inside responsive transcription factors and regulatory molecules [17]. At the molecular level the process of transdifferentiation for HSCs could be based on the finding that multipotent hematopoietic progenitors are primed for low-level transcription of nonhematopoietic loci, and that new microenvironment signals and the transcription factors balances could initiate gene expression of primed loci [17]. It can assume the same also for MSCs or other kinds of ASCs. In fact as BMHSCs, BMMSC are usually present in the BM stem cell niches under hypoxic conditions. Hypoxic conditions therefore influence MSCs proliferation and cell fate commitment, meaning that gradients of oxygen tensions influence the prolonged maintenance of a stem cell phenotype and pluripotency [57]. It has also been demonstrated that the culture of MSC under hypoxic conditions is accompanied by increased Oct4 expression and telomerase activity [57] which are involved in the maintenance of stemness. Hypoxic conditions induce the transcription factor hypoxia-inducing factor-*α* which can promote certain differentiation phenotypes in MSCs. Other lines of evidence, of microenvironment-dependant reprogramming of MSCs gene expression profile, come from studies on MSCs isolated from adipose tissue (ATMSCs). Thus, chondrogenic differentiation of ATMSC has been observed at enhanced levels under hypoxic conditions where osteogenesis is inhibited. In contrast, enhanced osteogenic differentiation of ATMSC can be induced under normoxia. 

Functional changes of MSCs under hypoxia also include increased secretory activity, that is, of vascular endothelial growth factor and interleukin-6 as well as mobilization and homing by the induction of stromal cell-derived factor-1 expression and the corresponding receptor CXCR4 [57]. In this context, MSC subpopulations displaying a high aldehyde-dehydrogenase activity have been reported with increased responsiveness to hypoxia, including an upregulation of Flt-1, CXCR4, and angiopoietin-2 [57]. Together, these findings further substantiate that BM hypoxic microenvironment and the chance in the microenvironment oxygen tension (from hypoxia to normoxia) contribute to the regulation of MSC function and fate [57]. 

Among the transcription factors known having essential roles in hematopoietic lineage decisions, there are GATA-1, Friend of GATA-1 (FOG-1), PU.1, *CCAAT/enhancer-binding protein* beta (C/EBP-*β*) and Pax. The zinc-finger transcription factor GATA-1 and its cofactor FOG-1 have been found to be essential for erythroid and megakaryocytic differentiation; the physical interaction between GATA-1 and FOG-1 is required for terminal erythroid and megakaryocyte maturation both *in vivo* and *in vitro *[58]. PU.1 is essential in the development of cells of the monocytic, granulocytic, and lymphoid lineage [58]. The cross-antagonism observed between GATA-1 and PU.1 and the relative abundance of each factor predict the lineage decision of a multipotent HSC. Moreover FOG expression is downregulated at the transcriptional level by C/EBP-*β*. This downregulation is a prerequisite for commitment to the eosinophil lineage [58]. Pax5 is another transcription factor whose expression in the hematopoietic system is restricted to cells of the B-cell lymphoid lineage. It was reported that pro-B cells derived from Pax5^−/−^ mice gave rise to several distinct lineages including macrophages, osteoclasts, and dendritic cells [58]; thus Pax5 normally represses alternative lineage programs. Another group of proteins which play a role in the regulation of cell identity, transcriptional memory, and plasticity is that encoded by the *Polycomb *(PcG) and *Trithorax* (trx-G) *Genes*. These proteins which regulate *Hox* genes expression pattern and determine segment identity in *Drosophila *are strongly involved in the regulation of hematopoiesis [17].

Less is known about genes and molecules involved in MSCs commitment and transdifferentiation. Among the transcription factors upregulated we note Cbfa 1 for induction of osteogenesis, Sox 9 for chondrogenesis, and PPAR*γ*2 for adipogenesis [56]. Satija et al. reported two other transcription factors governing osteogenic differentiation of MSCs: Osterix and Runx2 [59]. Several signalling pathways modulated by specific chemical compounds appear to be involved in the generation of myocardial cell types from MSCs, including the *bone morphogenic protein 4* (BMP4), *Wingless+ Int-1 *(Wnt), and *fibroblast growth factor 2* (FGF2) signalling, as well as inhibition of Wnt signalling by the factor *Dickkopf1 *(Dkk1) and the treatment with DNA-demethylating agent *5-azacytidin, *(5-aza) [50]. Recently it has been published that the transcription factor GATA-4 increases MSCs transdifferentiation into cardiac phenotype and enhances the MSCs secretome, promoting postinfarction cardiac angiogenesis [60]. What permits or restricts the access of transcription factors, coactivators, or constituents of transcriptional memory to genome regions and to particular genes within those regions? We know that nuclear programs consist of specific temporal, spatial, and geometric chromatin configurations. Epigenetic modifications (histone modifications, DNA methylation/demethylation, and ATP-dependent chromatin remodeling complex activation) that are generated in response to changing microenvironments, regulate these features of chromatin structure, support or not the opening of the chromatin, and are critical for the required nuclear reprogramming and thus transdifferentiation [61].

#### 1.4.2. MicroRNAs (miRNAs)

MicroRNAs (miRNA) are a class of noncoding RNAs which bind the 3′UTR of target mRNAs to mediate translational repression in cells. Many miRNAs are specifically expressed during hematopoietic lineage commitments [62]. miR-181, miR-223, and miR-142s were differentially or preferentially expressed in hematopoietic tissues; miR-142s expression was lowest in the erythroid and T-lymphoid lineages and highest in B-lymphoid and myeloid lineages; miR-223 expression was confined to myeloid lineages, with barely detectable expression in T- and B-lymphoid and in erythroid lineages [62]. Expression of miR-181, miR-223, and miR-142s was low in HSCs, suggesting that these miRNAs are also induced during lineage differentiation [62]. Moreover their differential expression in specific hematopoietic lineages suggested that they might influence hematopoietic lineage commitment and differentiation. In BMMSCs, miR-130 and miR-206 have been shown to regulate the synthesis of neurotransmitter substance P in human MSCs-derived neuronal cells [63]. 

#### 1.4.3. Cell Fusion rather than Transdifferentiation

It has been largely assumed that the nuclear reprogramming and transdifferentiation in response to environmental changes are the mechanism by which committed HSCs give rise to multiple cell types. However Terada et al. first reported the surprising results of an *in vitro* spontaneous cellular fusion between HSCs and totipotent ESCs in coculture [64]. Later, other reports contradicted the transdifferentiation phenomena of HSCs, showing that Purkinje neurons can fuse with BM-derived cells in both mice and humans [65]. The question is whether this *in vitro *fusion results in denying the transdifferentiation for *in vivo *HSCs switching. However, we must not forget that HSCs isolation protocols require manipulation that could expose highly enriched HSCs to concentrated pluripotent precursors types that might mediate cell fusion. In addition, one transplantation study in mice showed the 30%–50% efficiency of HSCs reconstitution of hepatocytes, which is far greater than the frequency (1 ~ 500.000) of HSC-ESC fusion observed [61]. This means that the lineage switching is not due to cell fusion but to transdifferentiation.

#### 1.4.4. BM Is a Source of Different Tissue-Specific Stem Cells

There is always the possibility that the BM hosts a variety of dedicated tissue-specific stem cells, such as muscle stem cells, neuronal stem cells, and hepatic progenitors, although there is, as yet, no evidence for the presence of these progenitors cells in the BM. It has also been postulated that a universal BMSC exists [23]. In this extreme view the various types of stem cells residing in the BM are considered to represent different states of a universal adult progenitor whose phenotype is defined by its local environment. These stem cells may move from one tissue into another via the circulation and may be more plastic in early than in more differentiated stages [23].

### 1.5. Negative Aspects of HSC and MSC Transdifferentiation

The transdifferentiation potential of HSCs and MSCs and their capacity for tissue renewal and damage repair have attracted much attention among biotechnologists and clinicians [66]. However some negative aspects must be considered. As Anderson has pointed out [67], there is a big difference between what cells normally do and what they can do if put in culture or if transplanted to a new location. From the perspective of cell therapy, however, it is what cells can do that may matter the most. In most reported cases, the phenotype of the donor-derived cells, that apparently switches their normal fate, was assessed by morphology and antibody staining, but rarely by function. Thus the cells may have acquired only a few of the characteristics of the new cell type but not any new functions. Cho et al. [68] reported that MSC-derived neurons exhibited synaptic transmission, but no evidence was provided that currents measured in cells were modulated by neurotransmitters. Similarly, Wislet-Gendebien et al. reported that MSC-derived neurons exhibit an evoked action potential, but a voltage spike induced only modest membrane depolarization [69]. Moreover in MSC-derived cardiomyocytes, the expression of cardiac markers such as cardiac *α*-actin, the *Desmosomal Type Junction Proteins Desmoglein 2 *(Dsg2), *Desmocollin 2* (Dsc2), desmoplakin and plakophilin 2, and the junction protein myozap has not been found [50]. This is a major problem as all these molecules are known to be important for the formation of the composite junctions in the intercalated disk [50]. Even if they are the stimuli of the microenvironment to direct transdifferentiation, it is also possible that the differentiation is directed towards unwanted tissues. Recently BMMSCs injected into rat hearts were shown to differentiate into bone tissue and to drive its calcification [70]. 

A negative aspect of HSCs transdifferentiation is their contribution to BM neovascularization which represent a problem in those cancers which home and expand in the BM. Ria et al. demonstrated that in patients with multiple myeloma (MM), but not in those with *Monoclonal gammopathy of undetermined significance *(MGUS), *hematopoietic stem and progenitor cells* (HSPCs) differentiate into cells with endothelial features, contributing to the neovessels wall building together with MM endothelial cells (MMECs) [71]. Moreover in patients with MM, BM macrophages and mast cells transdifferentiate in to endothelial cells thus contributing to vasculogenic mimicry [72, 73]. We know that BM neovascularization contributes to MM progression [74]. Finally, we assume that the mechanism of transdifferentiation could be congenial not only to ASCs or to their precursor cells, but also to their tumor staminal counterparts: the *cancer stem cells* (CSCs). In gliomas transdifferentiation of CSCs into vascular mural cells contributes to tumor neovascularization [75]. Assuming the existence of hematopoietic and mesenchymal cancer stem cells residing in the BM, it can equally suppose their ability to migrate through the bloodstream and reach new districts where, in response to new microenvironment stimuli, they could transdifferentiate in to several tumor cell types generating metastasis and new tumors.

### 1.6. Clinical Applications of BM HSC and MSC

BMSCs are an attractive source of cells for therapy, especially in view of the recent claims that they are remarkably plastic in their differentiation potential when exposed to new environments. 

Transplantation of BMSCs is traditionally used for haematological diseases, but there are increasing numbers of clinical trials using BMSCs for the treatment of nonhematological disorders. Xu and Liu resumed the studies carried out in animal models and in humans underlying the therapeutic potential of BMSCs in liver diseases [7]. This potential consisted in the restoration of liver function and liver mass, supply of growth factors, antifibrosis, and gene therapy. Recently the clinical trials involving BMSCs transplantation for the therapy of myocardial infarction [11] and *spinal cord injury* (SCI) [76] have been resumed. Another clinical application of BMSCs could be the treatment of xerostomia due to head and neck irradiation for cancer therapies and in Sjogren's syndrome and reestablishing of the salivary gland functions [77]. 

Among BMSCs, HSCs are the only stem cells being routinely used in the clinics [78]. They constitute only a small fraction of BM population (1 in 10^4^ to 1 in 10^8^ of BM nucleated cells), but the stimulation with mobilizing agents, including cytokines such as *Granulocyte Colony-Stimulating Factor *(G-CSF) alone or in combination with *granulocyte-macrophage colony-stimulating factor *(GM-CSF) and/or other agents, dramatically increases the release of HSCs from BM to PB [78]. HSCs are primarily used in the treatment of patients with haematological malignancies. During the course of treatment, patients' cancerous cells are first destroyed by chemo/radiotherapy and subsequentally replaced with autologous PB/G-CSF HSCs collected prior to the treatment, and reinfused into the patients, or with BM or PB/G-CSF transplant from a human-leukocyte-antigen-(HLA)-matched donor [78]. Allogenic BM transplant have also been used in the treatment of hereditary blood disorder including aplastic anemia, *β*-thalassemia, Wiskott-Aldrich syndrome, and *severe combined immunodeficiency* (SCID) as well as in metabolism errors as Hunter's syndrome and Hurler's syndrome [78]. HSCs transplants are also used as a therapeutic strategy against various types of solid tumors [78]. 

MSCs have become a recent focus of interest for cellular therapy in tissue regeneration. Wound healing studies have focused on MSCs as the cell population within the BM that can contribute to cutaneous regeneration [79]. Experiments with diabetic murine models have been particularly useful in assessing the clinical utility of MSCs in wound repair. Promising findings in animal models have led to a very limited number of human trials examining the effects of autologous MSCs on chronic wounds. Injection of primary BM cells into the wound edge followed by topical application of cultured MSCs, resulted in the complete closure of three chronic wounds which had failed traditional therapy including autologous skin grafting [79]. Dash et al. conducted a randomized trial investigating the use of autologous MSCs expanded in culture and injected intramuscularly into the wounds edges of 24 patients with nonhealing ulcers secondary to diabetes or vasculitis. Ulcer size in the MSC-treated group decreased 73% [80]. MSCs enhace wound healing not only by differentiating into epidermal cells, but also into vessel forming endothelial cells contributing to neovascularization, necessary to supply oxygen and nutrients to the damaged tissue [80]. Another clinical application of MSCs would be to exploit their osteoblastic potential for treating bone disorders as in osteogenic imperfecta (OI). After a first demonstration of the potency of MSCs to differentiate into functional osteoblasts in a mouse model of OI, following a first BM transplantation, MSCs were used in children with type III OI [81]. These children showed improved growth and even low osteopoietic engraftment of MSCs was evident [81]. Recently the clinical trials have been also resumed involving BMMSCs in the treatment of neurological diseases such as traumatic brain injury, *spinal cord injury* (SCI), parkinson's disease, multiple sclerosis and amyotrophic lateral sclerosis, but these current data do not support the possibility that most of the reported effects occur as a result of direct transdifferentiation and cell replacement [82]. Some clinical trials have been performed with MSCs to treat heart damage. A Chinese group performed intracoronary short injection of autologous cultured BM cells after acute myocardial infarction and for the treatment of chronic ischemic cardiomyopathy [83]. The authors found improved cardiac function in patients the receiving cells [83]. A group from Greece performed a similar study and found the procedure to be safe and contributing to regional regeneration of myocardium [84].

#### 1.6.1. Advantages and Disadvantages of BM HSCs and MSCs Therapy

BMSCs have a major advantage over stem cells from other organs: they are well defined, easy to isolate, and can be injected systemically reaching other tissues through the bloodstream. Thus they are more suitable than other kinds of stem cells for the therapeutic use. The advantage of BMSCs isolation compared to other types of ASCs (neuronal, heart, and kidney stem cells) resides in the properties of mobilization and homing. In fact BMSCs migrate from their BM niche to PB and then return to a new site in the BM. Presumably, some of the mechanisms that regulate stem cell trafficking are the same that regulate homing and lodging of BMHSCs during transplantations. The comparison between the clinical use of BMHSCs and BMMSCs shows a greater number of applications for the latter than the former. Although preclinical studies have demonstrated the plasticity of both BMHSCs and BMMSCs, the majority of clinical trials see the use of BMHSCs for the treatment of hematological malignancies in which the capacity of HSCs to reconstitute the hematopoietic system of the patient rather than the transdifferentiation potential is exploited. On the contrary BMMSCs applicability in therapy exploits their potential to differentiate into different cell types and this explains their increased use in the clinical trials of various diseases. However in the treatment of haematological diseases BMHSCs show many advantages compared to other sources of HSCs such as *Cord Blood Stem Cells* (CBSCs). It was learned that one umbilical cord contains an adequate number of HSCs for a successful engraftment only in low body weight patients (up to 40 kg) to reconstitute their immune system. The total number of cells, comprising hematopoietic progenitors, collected from one umbilical cord is significantly lower (roughly 5 × 10^6^) than from BMSCs or from PB after BMSCs mobilization (roughly 1 × 10^8^) [85]. Furthermore obese patients can be treated by BM transplants, as multiple units of cord blood are required [85]. BMSCs and BMSCs mobilized into the PB show also some disadvantages: they are mostly nondividing cells and have, respectively, 3 times and 6 times less repopulating cells than CBHSCs [85]. Another disadvantage of using autologous BMHSCs in cancer therapy is that cancer cells are sometimes inadvertently collected and reinfused back into the patients with the HSCs. One team of investigators finds that they can prevent reintroducing cancers cells by purifying cells and preserving only cells are CD34+, Thy-1^+^ [86]. As BMHSCs, BMMSCs show minor proliferative capacity, life span, and differentiation potential compared to MSCs from birth-associated tissues such as placental andumbilical cord MSCs [57]. BMMSCs show an important advantage compared to BMHSCs: immunosuppressive properties. MSCs infusions in autologous or allogenic HSC transplantation could reduce the risk of graft failure and the incidence of acute *graft-versus-host disease* (GvHD) [81]. In fact MSCs have been shown to interact with many cell types of the immune system affecting both innate and adaptive immunity by inhibiting proliferation, differentiation as well as the function of monocytes, dendritic cells, NK cells, T cells and B cells. However it has been reported that MSCs may also act as non-professional antigen-presenting cells and that they express toll-like receptors and thus can respond to pathogen-associated molecules that stimulate immunoresponse. Thus the exact mechanisms how MSCs regulate the immune system are still not completely understood. However for their immunosoppressive properties, MSCs were found to help with tumor development *in vivo *promoting the development of a permissive stroma for the tumor, as was demonstrated in MM [81]. Another advantage of BMMSCs therapy is that they secrete many growth factors stimulating hematopoiesis, provide a scaffold for hematopoiesis, and support primitive progenitors cells *in vitro *[81]. Thus MSCs improve HSCs engraftment [81].

#### 1.6.2. Potential Bottlenecks in BM HSC and MSCs Therapeutics

Although BMHSCs and BMMSCs belong to the most intensely studied stem cell types in cell therapy, comparison of existing preclinical and clinical data is hampered by a poor standardisation and harmonisation concerning protocols for isolation, expansion, and delivery. 

As with BMHSCs, BMHSCs mobilized into PB contain a mixture of hematopoietic stem cells, progenitor cells, and other kinds of cells. Consequently, the resulting cell preparation that is infused back into patients is not a pure HSCs preparation, but a mixture of HSCs, progenitors, and various contaminants, including T cells and in the case of autologous graft from cancer patients quite possible tumor cells as described previous. BMHSCs normally passed through a device that enriches cells that express CD34+, a marker of both stem and progenitor cells. The use of highly purified HSCs as graft is rare [87]. The main problem associated with clinical use of highly purified HSCs is the additional labor and costs involved in obtaining highly purified cells in sufficient quantities. 

Efficient expansion of HSCs in culture remains one of the major goals despite their ability to self-renew. Attempts to expand HSCs in tissue culture with known stem-cell stimulators (growth factors and cytokines) have never resulted in a significant expansion of HSCs. Rather, these compounds induce many HSCs into cell division that are always accompanied by cellular differentiation [88]. Compared to HSCs, MSCs are strictly anchorage dependent and therefore need a surface to attach and proliferate. Simple ways for the cultivation of adherent cells in larger quantities are monolayer culture flasks such as roller bottles or multiple plate vessels. It has been shown that in static monolayer cultures MSCs proliferate slower and the differentiation potential is affected as well [89]. The use of a bioreactor is an alternative to the expansion in flasks. Bioreactors provide conditions similar to the *in vivo* situation of the cells, including advantages such as efficient nutrient supply, waste removal, minimal shear stress, and the possibility to control the cultivation via online measreuments of critical values [89]. 

Another bottleneck of stem cells therapeutics is the way of administration and the cells delivery. In transplants HSCs are generally infused intravenously. For MSCs the researchers have tried to optimize the delivery. In the treatment of cutaneous wounds, most studies have utilized the technically simple method of injecting a cell suspension intradermally into or around the wound defect; however the true therapeutic potential of MSCs appears to be limited due to poor engraftment efficiency and cell retention at the wound site. A fibrin spray system, to topically administer autologous MSCs to nonhealing lower extremity wounds, has been used in human subjects. Stem cells were found to survive within the fibrin layer and migrate into the wound tissue [79]. Hydrogels are synthetic biomaterials that emulate the hygroscopic nature of extracellular matrix making them an ideal vehicle for MSCs delivery [79]. A novel collagen-pullulan hydrogel that is noncytotoxic and provides protection from oxidative stress was recently described. MSCs seeded and cultured in this hydrogel significantly accelerated wound closure and improved quality of cutaneous regeneration when compared to intradermal injection strategies [79]. 

Previous examples demonstrate that questions needs to be answered in applying BMSCs in therapeutics, such as: (i) when to use pure BMSCs preparation in transplants; (ii) whether markers of HSCs, currently known, can distinguish them from their tumor counterparts; (iii) how to improve BMSCs expansion in culture without altering their stemness and differentiation potential; (iv) how to determine the most efficient method of administration of BMMSCs and how to optimize their delivery. Answering these questions will lead to a better standardization of methods and protocols used in the manipulation of BMSCs and to the overcoming of the most common bottlenecks in BM HSCs and MSCs therapeutics.

### 1.7. Conclusions and Future Perspectives

The therapeutic potential of BMSCs as powerful tools in tissue regeneration and engineering has been recognised, and intense efforts are ongoing to harness and direct HSCs and MSCs plasticity. However before HSCs and MSCs are currently used therapeutically in patients with degenerative disorders of the liver, heart, or brain, the properties of such cells must be well characterized, the functionality proved, and the potential risks of their use well defined. Understanding the molecular mechanisms underlying cell fate switching of BMSCs will be an essential contribution to ensuring their safe use in regenerative medicine. Moreover, even if the transdifferentiation events described in most of these studies were rare under physiological conditions, in the future, it will most likely be possible to transplant genetically modified stem cells carrying genes critical for transdifferentiation into desired cell populations. Finally pharmacologic molecules would also be used to directly influence the trans- or redifferentiation potential of ASCs, both prior and after their administration into patients.

## Abbreviations

BMSCs: Bone marrow stem cells
HSC: Hematopoietic stem cell
MSC: Mesenchymal stem cell
CLP: Common lymphoid progenitor
CMP: Common myeloid progenitor
GMP: Granulocyte/macrophage progenitor
MEP: Megakaryocyte erythrocyte progenitor
T: T-lymphocyte
B: B-lymphocyte
MAC: Macrophage
G: Neutrophil granulocyte
EOS: Eosinophil
EB: Erythroblast
MEG: Megakaryocytes.
